# Rules of the road: how to turn off kinesin motors

**DOI:** 10.1042/BCJ20253135

**Published:** 2026-01-28

**Authors:** Zhenyu Tan, Alex Missman, Michael A. Cianfrocco

**Affiliations:** 1 Life Sciences Institute, University of Michigan, U.S.A.; 2 Department of Biophysics, University of Michigan, U.S.A.; 3 Department of Biological Chemistry, University of Michigan, U.S.A.

**Keywords:** ATPase, cryo-electron microscopy, intracellular transport, kinesins, microtubule, structural biology

## Abstract

Intracellular organization is crucial for supporting cell function in an ever-changing environment. The eukaryotic microtubule cytoskeleton and its associated motor proteins are the vast molecular highways and motor vehicles that connect, position, and transport cellular cargoes, ranging from the cell nucleus to vesicles to mitotic spindles. The kinesin superfamily of motor proteins carries out a diverse array of functions and is thus a key player in these processes. While the mechanochemical cycle of kinesins has been extensively studied, mechanisms of kinesin activation and inhibition are not well understood. Over the past five years, several publications have significantly advanced our understanding of kinesin regulation, showing how inesin motors are turned off via autoinhibition and kinesin-binding protein. In this review, we will delve into these recent findings to introduce some ‘rules of the road’ in a model that captures the complexities of kinesin regulation.

The discovery of conventional kinesin (kinesin-1) from squid axoplasm approximately four decades ago marked a significant advancement in the field of motor proteins [1,2]. Since then, numerous additional kinesin motor proteins have been identified, each employing a unique function. In humans, there are a total of 45 kinesin motor proteins, which can be grouped into 15 distinct families from kinesin-1 to kinesin-14 [3–6]. Each kinesin family exhibits unique structural features and biophysical properties that allow them to perform specific functions within cells.

Kinesins share a P-loop ATPase catalytic core, better known as the motor domain, which generates mechanochemical force. Kinesin motor domains have been extensively studied and reviewed elsewhere [3,4,7–9]. Based on their cellular roles, kinesins can be generally classified into two categories: transport kinesins and mitotic kinesins [3]. Transport kinesins, such as kinesin-1, -2, and -3, are predominantly plus-end–directed motors that collaborate with dynein and myosin to deliver vesicles, organelles, and macromolecular cargo to precise subcellular destinations [7]. In contrast, kinesin-4, -5, -6, -7, -8, -10, -12, -13, and -14 function primarily during mitosis to organize the spindle and ensure accurate chromosome segregation [4,10,11]. Within this latter group, kinesin-14 motors are unique in moving toward the microtubule minus end [7], whereas kinesin-8 and kinesin-13 regulate microtubule dynamics through promoting depolymerization [7].

The goal of this review is to focus on how kinesin motors are parked and put into an ‘off’ state through *cis-* and *trans-*inhibition. *Cis*-inhibition represents the best-understood mode of kinesin inhibition, whereby full-length kinesin motors adopt autoinhibited states to slow ATPase activity and microtubule binding. Autoinhibition is important for the biological function of presumably all kinesins, such as in kinesin-1 [12–15] and kinesin-3 [16,17]. For the sake of this review, we will focus our discussion on the most well-studied autoinhibition of kinesin-1.

*Trans*-inhibition is a newly discovered form of kinesin regulation that we and others have recently characterized, in which kinesin-binding protein (KIFBP) binds directly to the kinesin motor domain to prevent microtubule binding. We will discuss both modes separately in the text and end the review with a model incorporating both kinesin regulation modes.

## Part I: *cis*-inhibition of kinesin via autoinhibition

The study of how motor proteins are spatially controlled led to the model that the majority of, if not all, cytoskeletal motors (kinesins, dyneins, and myosins) are regulated through autoinhibition. In the absence of cargo, motor proteins exist in the autoinhibited state, which restricts their activity and prevents aberrant cargo transport—much like the hand brake on a car prevents a runaway vehicle. Conversely, cargo binding will lead to activation, enabling the motor to drive. Recent structural analysis of cytoplasmic dynein-1 shows that it adopts an autoinhibited conformation called ‘phi particle,’ in which the motor domain locks itself in a low microtubule affinity state [18,19]. This autoinhibited conformation can be relieved through the co-operative action of cargo adaptors, dynactin, and Lis1, allowing the formation of an active dynein complex [20–23]. Similarly, the structure of autoinhibited conformation was recently determined by cryo-electron microscopy (cryo-EM) for several myosin families [24,25]. In contrast, while kinesin was discovered nearly 40 years ago, our understanding of the structure and mechanism regarding kinesin inhibition and activation lags behind that of dynein and myosins.

Among the kinesin superfamily, kinesin-1 serves as a model system to understand the regulation of kinesins via autoinhibition. Kinesin-1 can exist in two cargo-free forms: a homodimeric form that includes two motor domain-containing kinesin heavy chains (KHC) and a heterotetrameric form that contains two heavy chains and two kinesin light chains (KLCs) [26–28]. The KHC can be divided into three parts: the N-terminal motor domain, a stalk consisting of a series of coiled-coils, and the disordered C-terminal tail [29]. The KLC contains two elements: an N-terminal coiled-coil that associates with the KHC and the C-terminal tetratricopeptide repeat (TPR) domain, which functions as a cargo-binding platform [30–33].

The kinesin-1 motor adopts a folded, compact conformation in the autoinhibited state. Since the discovery of kinesin-1, several groups examined the purified kinesin-1 from various sources by electron microscopy (EM) and found that kinesin-1 can either adopt an extended, almost linear conformation or a folded, compact conformation [28,29]. Length measurements showed that the length of folded kinesin-1 is about half the length of extended kinesin-1, suggesting that kinesin-1 can fold in half via the stalk. Later, sedimentation and hydrodynamic analysis on kinesin-1 showed that the conformation transitioned from a compact to an extended state under increased salt concentration [34]. Subsequent enzymatic assay revealed that kinesin-1 exhibits minimal microtubule-stimulated ATPase activity in its compact form. In contrast, relieving kinesin-1 from its compact state by attaching it to plastic beads or a glass surface showed a significantly increased ATPase activity, which indicates a model in which the compact form of kinesin-1 is the inhibited state, while its extended form is the activated state [35,36].

Subsequent work identified a C-terminal region in the heavy chain required to maintain the inhibited form of kinesin-1 [30,37]. To test this hypothesis, a series of C-terminal truncated KHC constructs were made and assayed for their ability to undergo the salt-dependent conformation transition and microtubule-stimulated ATPase activity. The results showed that the kinesin-1 exhibited increased ATPase activity and a more extended state as the C-terminus was progressively truncated, indicating the loss of autoinhibition [37]. Later, a 15-amino-acid-long sequence motif containing conserved residues isoleucine (I), alanine (A), and lysine (K) (IAK motif) in the C-terminal tail was shown to be critical for autoinhibition [38]. X-ray crystallography of an IAK-containing tail peptide bound to kinesin motor domains revealed that the peptide occupies the interface between the two motor domains but does not sterically block microtubule binding. The core IAK motif and adjacent residues make contact with both motor domains, thereby restricting their relative movement. This steric constraint likely prevents conformational changes, such as neck-linker undocking, which are required for the kinesin-1 mechanical stepping cycle [39]. Thus, the IAK motif was believed to be the critical element in maintaining kinesin-1 autoinhibition.

Structural analysis of KHC suggested the molecule folds in a long series of coiled-coil regions and an unstructured tail domain. Based on coiled-coil predictions, a low-confidence prediction between coiled-coils 2 and 3 led to the proposal that the hinge allowing KHC to bend in half sat between these two coiled-coils. The importance of this proposed hinge was verified by data showing that removal of this hinge and fusion of the adjacent coiled-coils increased the microtubule-stimulated ATPase activity and motility in a single-molecule assay [35]. Based on this evidence, removing C-terminal coiled-coils and the tail generated an activated version of kinesin-1 that contained residues 1–560 [35]. This truncated kinesin-1 was called ‘K560’ and was commonly used for *in vitro* and cell-based assays as a paradigm of active kinesin-1 [40–44].

Beyond KHC, the KLCs also regulate kinesin-1 motility. KLCs simultaneously serve to further stabilize the autoinhibited state and allow specific cargo binding. Early imaging analysis showed that co-transfection of KLC with KHC in COS7 cells strongly decreased the localization of kinesin-1 on microtubules, suggesting that KLCs inhibit kinesin-1. This was supported by the microtubule cosedimentation assay, in which the interaction of KLC with KHC reduces the ability of KHC to bind microtubules [30,45]. Recently, *in vitro* single-molecule assays on kinesin-1 heterotetramers further confirmed that the KLCs suppress motility [46]. However, it remained unclear from a structural perspective how KLCs inhibit the kinesin-1 activity and whether it will promote a new structural state different from the inhibited heavy chain dimer.

In addition to maintaining KHC in an off state, KLCs also function as cargo binding platforms, physically linking KHC to various cargoes. Cargoes, such as SifA-kinesin-interacting protein (SKIP), JNK-interacting protein 1 (JIP1), and Nesprin4, rely on either W-acidic or Y-acidic motifs to dock them onto the inner concave surface of the TPR motif in KLCs [33,47,48]. Other cargoes that do not contain the above motifs utilize a different way to interact with KLCs. JNK-interacting protein 3 (JIP3), for example, cross-links two TPR domains through its leucine zipper [32]. Although all the above cargo physically interacts with KLCs, only the combined presence of a KLC cargo (e.g. Nesprin4) with microtubule-associated protein 7 (MAP7) has been shown to activate kinesin-1 motility in a purified *in vitro* system [46], suggesting that cargo binding to TPR may not be sufficient to relieve kinesin-1 from the autoinhibited state. Therefore, the detailed molecular mechanisms of cargo-mediated activation remain open.

Despite this prevailing model that autoinhibited kinesin-1 folds back via a hinge in a head-to-tail fashion to allow the IAK motif to inhibit the motor domain, recent data have challenged this model: mutating the IAK motif or deleting the hinge does not activate kinesin-1 [46]. Using *in vitro* single-molecule motility assays, McKenney and co-workers assayed the motility of all three isoforms of human kinesin-1, showing little landing and processive motility on microtubules. Surprisingly, mutating IAK to AAA did not result in a substantial increase in processive motility but only increased the landing rate. Similarly, the removal of the hinge caused the aggregation of kinesin-1 and showed no increase in the frequency of processive motility. The contradictory data regarding the IAK motif and hinge indicate that the autoinhibition mechanism involved more than these two elements.

### New structural insights into the autoinhibition mechanism of kinesin-1

Recent advances in protein structure prediction have helped to shed light on the architecture of autoinhibited kinesin-1. To reveal the coiled-coil domain conformation in autoinhibited kinesin-1, Dodding and co-workers employed AlphaFold2-Multimer [49,50] to predict the structure of the kinesin-1 stalk from coiled-coil 1 to coiled-coil 4 [51]. The predicted stalk structure resembles four coiled-coil domain arrangements. However, it revealed an unexpected location regarding the hinge. Conventional coiled-coil prediction suggests that the hinge sits between coiled-coil 2 and coiled-coil 3. Surprisingly, this original hinge region was predicted to be a short, rigid tetrameric coiled-coil. Instead, the stalk folds back via an unexpected coiled-coil breaking point within coiled-coil 3, which they named ‘elbow.’ To resolve the stalk conformation in the kinesin-1 heterotetramer, they predicted the structure of the KLC coiled-coil bound to the stalk. The binding interface of KHC and KLC was predicted to be the dimer of antiparallel trimeric coiled-coil and is consistent with the previously reported location.

To validate the predicted stalk conformation, Dodding and co-workers expressed and purified kinesin-1 and used negative stain EM to assess its conformation. The purified kinesin-1 heavy chain sample was subjected to size-exclusion chromatography (SEC), and two peak populations were identified. The sample corresponding to each peak was cross-linked and analyzed by negative staining EM. Samples from the first peak exhibit a long, extended conformation, ∼80 nm long. In contrast, samples from the second peak showed a more compact, V-shaped conformation with a length of ∼40 nm, corresponding to the autoinhibited conformation. Particles from the autoinhibited conformation were picked and processed to generate the 2D class averages. The class average revealed two globular motor domains and a rod-shaped stalk, which agrees with the projection image generated from the predicted stalk structure, suggesting that the kinesin-1 stalk folds back via the elbow. To further validate the presence of the elbow and its role in mediating the folding of the stalk, a kinesin-1 mutant with the elbow deleted was made and tested. This mutant lacked the second SEC peak corresponding to the folded species and displayed only an extended morphology under negative-stain EM, demonstrating that removal of the elbow abolishes the ability of the stalk to fold back and form the autoinhibited conformation.

Taken together, this work proposed a new mechanism of stalk folding in the autoinhibited kinesin-1 and suggested the real hinge location where kinesin-1 folded back. However, there was still limited understanding of the autoinhibited conformation due to the lack of experimental structural data beyond negative stain EM.

To provide a more detailed view of autoinhibited kinesin-1, work from our lab combined negative staining EM, structure prediction, and cross-linking mass spectrometry (XL-MS) to generate the complete architecture of autoinhibited kinesin-1 [52]. Our work showed that the widely accepted model of kinesin-1 autoinhibition, where kinesin-1 folded via the middle hinge to allow the tail motor domain interaction, is not completely accurate in describing the kinesin-1 autoinhibition. Based on our data, we proposed a new model of kinesin-1 autoinhibition where kinesin-1 utilizes a hierarchical folding mechanism to scaffold itself into the inhibited state.

First, our data now suggest the actual hinge position of kinesin-1 to be in the coiled-coil 3, which divides the coiled-coil 3 into CC3a and CC3b. The previously assumed hinge between CC2 and CC3 is predicted to be a structured tetrameric coiled-coil. This discovery reconciles conflicting data concerning hinge deletion and kinesin-1 activation. Our data and those from Dodding and co-workers have both demonstrated that removing the real hinge leads to the opening up of kinesin-1 and enhancement of the landing rate in single-molecule assays.

**Figure 1:**
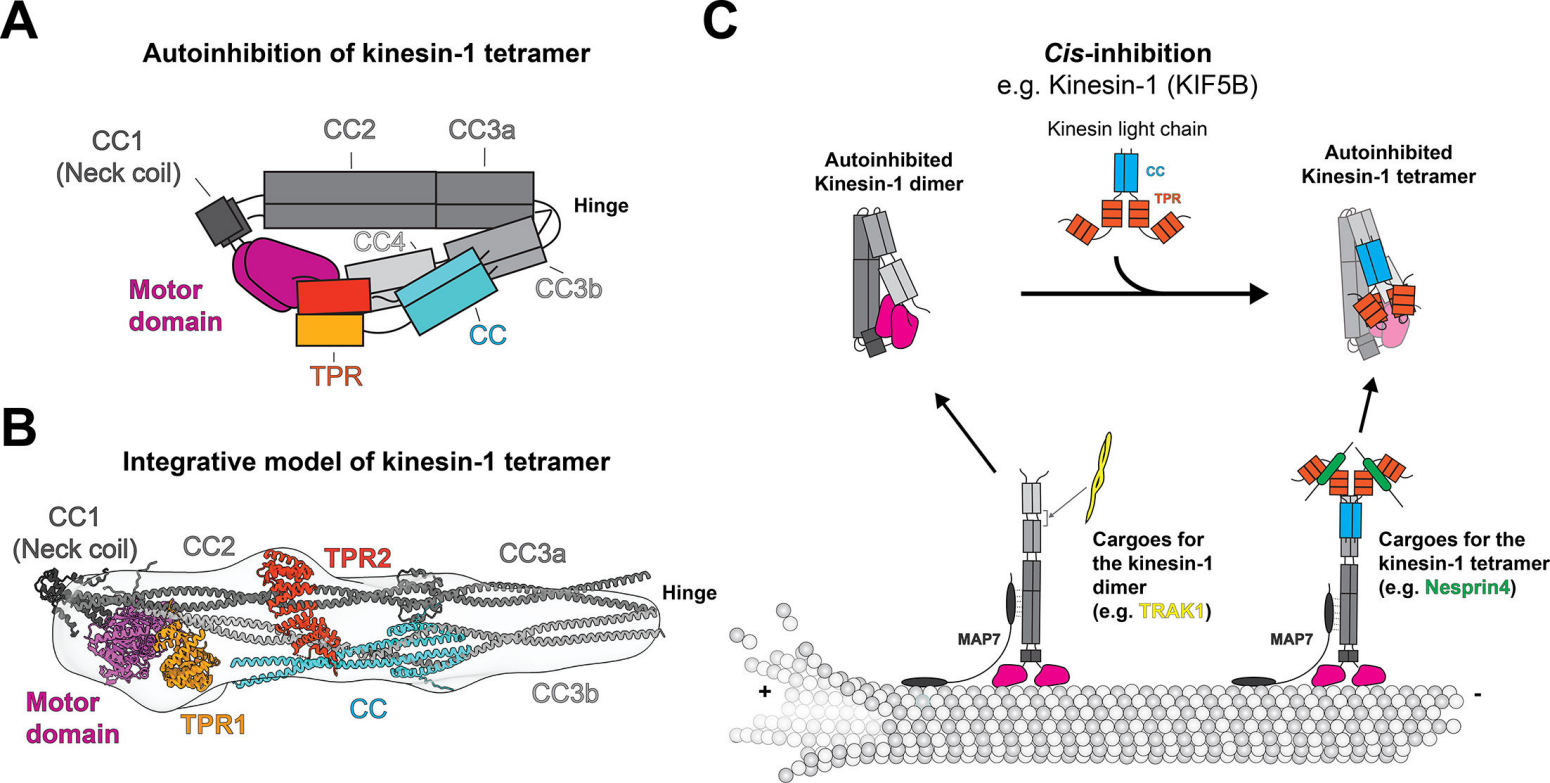
Kinesin autoinhibition and activation. (**A**) A cartoon schematic depicting the autoinhibited kinesin-1 tetramer. (**B**) An integrative structural model of the autoinhibited kinesin-1 tetramer, using AlphaFold2, cross-linking mass spectrometry, and negative stain EM. (**C**) A model of kinesin-1 regulation, which undergoes *cis*-inhibition.

Second, we proposed that the two motor domains scaffolded back, and one of them docked onto the CC2 in a manner that sterically blocked the kinesin motor microtubule-binding surface. Positioning the motor domain along CC2 also enables the C-terminal of CC4 and tail to bind between the motor heads and stabilize the autoinhibited state (Figure 1A). Our model is consistent with the observation that a CC2 truncated kinesin-1, 1–400, is more active than the original 1–560 truncation [41,52], which still preserves the motor to CC2 cross-links.

Third, our data emphasized the importance of the whole tail domain, not just the IAK motif, in stabilizing the autoinhibited conformation through association with the motor domain. In our single-molecule motility assay, we showed that mutating the IAK motif only results in a moderate increase in the landing rate compared with the tail domain truncated kinesin-1, suggesting that mutating IAK is not sufficient to activate kinesin-1. In supporting this, we observed that kinesin-1 with IAK mutation mainly exhibits a compact conformation under EM and mirrors the cross-linking pattern of wildtype (WT) kinesin-1. Taken together, we proposed that the tail domain, as a whole, is important for maintaining the autoinhibited conformation of kinesin-1. This hypothesis agrees with the structural model of autoinhibited kinesin-1, in which the tail domain lies very close to the motor domain, and its upstream CC4 makes extensive cross-links with the motor domain.

Finally, our data demonstrate that the KLC stabilizes the folded conformation of the KHC rather than inducing a new structural state. Our XL-MS showed that the kinesin-1 heterotetramer exhibits the same folding pattern in the heavy chain compared with the kinesin-1 homodimer. Furthermore, the predicted structure of kinesin-1 heterotetramer also supports that the KLC did not change the folding pattern of the heavy chain (Figure 1B).

Following our work, a similar integrative structural biology approach was applied to studying the autoinhibition of KIF5A, one of the kinesin-1 isoforms in humans. KIF5A has a longer C-terminal tail than the other two kinesin-1 isoforms (KIF5B and KIF5C), and its autoinhibition mechanism remains unclear compared with KIF5B and KIF5C. Furthermore, KIF5A is neuronal specific and mutations can cause neuronal disease [14,53]. To probe the structure of autoinhibited KIF5A, Peckham and co-workers utilized negative-stain EM, XL-MS, and AlphaFold2 structure prediction to determine the molecular architecture of the autoinhibited KIF5A [54]. KIF5A exhibits the same autoinhibition pattern as KIF5B and KIF5C. Interestingly, its long C-terminal tail was not detected to form cross-links with other parts of the molecule, and a mutation that altered the sequence of the tail did not induce the structure changes of the autoinhibited conformation. This work, in combination with the above two studies, showed that kinesin-1 family isoforms generally share modes of autoinhibition.

In summary, our work, together with others, integrates AlphaFold structure prediction, XL-MS, and single-particle EM to propose a refined model for autoinhibited kinesin-1. Our model suggests that kinesin-1 adopts a hierarchical folding architecture in the off-state, in which multiple layers of intramolecular interactions co-operate to stabilize the compact, autoinhibited conformation. Definitive, high-resolution structures of the inhibited complex will be crucial to resolve the underlying interaction network in molecular detail.

Notably, a recent ~8 Å cryo-EM reconstruction of the autoinhibited kinesin-1 heterotetramer revealed a self-folded stalk and an unexpected arrangement of the KLCs [55]. This structure provides an important foundation for future efforts aimed at achieving near-atomic resolution of the inhibited complex. Such structures will not only validate and refine current models but also illuminate how regulatory partners and cargo binding remodel kinesin-1 to initiate transport.

## Future directions

The recent structural data on kinesin-1 autoinhibition allow us to propose a more detailed model of kinesin-1 activation (Figure 1C). In the inhibited state, the kinesin-1 heavy chain can adopt an autoinhibited folded conformation as either a homodimer or a heterotetramer. Notably, KLC does not impart global rearrangements of KHC in the heterotetramer. Furthermore, from either homodimeric or heterodimeric KHC, cargo can activate kinesin motility in the combined presence of MAP7. Even though most KHC is likely in the heterotetramer form in cells, Trafficking kinesin-binding protein 1 (TRAK1) is a mitochondrial cargo adaptor that can activate kinesin-1 motility without light chains [56]. Despite being able to bind and open KHC, TRAK1-driven kinesin-1 motility is further enhanced by the presence of MAP7 [ 57]. In a manner analogous to KHC homodimers, heterotetramers of KHC:KLC also require both a cargo and MAP7 for motility [46].

The multi-component activation of kinesin-1 motility is consistent with the current structural model of kinesin-1 autoinhibition: activation requires the ‘opening up’ of KHC by competing with multiple interacting regions of KHC that keep motors in an off state. Future work will help to understand the diversity of kinesin-1 activation mechanisms for either homodimeric or heterodimeric kinesin-1 motors involving cargo adaptors, such as Fasciculation and elongation protein zeta-1 (FEZ1) [58], Huntingtin-associated protein 1 (HAP1)/Glutamate Receptor Interacting Protein 1(GRIP1) [59], or the JIP family of cargo adaptors [60].

Despite advances in our understanding of autoinhibition, there are several unknowns regarding the role of tail and light chains that we will highlight below.

The detailed conformation of the tail domain and its role in maintaining the autoinhibited kinesin-1 requires further study. Early biochemical data showed that a single tail domain binding to the kinesin-1 motor dimer is sufficient to block microtubule-stimulated ADP release and ATPase activation [38]. The crystal structure of the kinesin-1 motor dimer subsequently revealed the IAK motif ‘zipping’ the motor heads into a constrained orientation, locking the neck linkers and suppressing ADP release [39]. Our autoinhibition model is consistent with these findings, demonstrating that the tail remains closely associated with the motor domains, as supported by extensive CC4–motor cross-links. In addition, our analysis indicates that the entire tail contributes to maintaining the inhibited conformation. However, neither our XL-MS data nor others’ studies have detected direct cross-links between tail residues and the motor domains, likely reflecting the low lysine content of the tail rather than the absence of interaction. Defining the precise conformation of the tail in the autoinhibited complex therefore remains a critical challenge.

Recent cryo-EM structures revealed tail-bound kinesin-1 motor dimers on microtubules in multiple nucleotide states [61]. Consistent with crystallographic data, the tail cross-bridges the two motor heads but does not alter the conformation of the microtubule-bound head across nucleotide states. Based on these observations, they proposed that the tail primarily inhibits forward stepping rather than blocking microtubule-induced structural transitions per se, thereby preventing cargo-free kinesin-1 from productively engaging and moving along microtubules. However, how the tail domain functions in the context of full-length autoinhibited kinesin-1 remains unclear, which requires further structural study to map its location in autoinhibited kinesin-1.

Beyond the tail, a complete understanding of kinesin-1 autoinhibition also requires consideration of KLC, which contributes an additional layer of regulation and enables cargo-dependent activation. Our data showed that kinesin-1 light chains do not prompt the formation of a new structural state of the heavy chain. Furthermore, the light chain coiled-coil domain alone was sufficient to maintain the kinesin-1 heterotetramer in the inhibited state [46]. In contrast, the role of the KLC TPR domain in autoinhibition remains less clear. Based on low-resolution EM density, we proposed two possible TPR positions: (1) adjacent to the motor domains and (2) along the stalk (Figure 1B). However, many of the observed XL-MS cross-links between the TPR and heavy chain exceed the expected distance constraints for BS3, suggesting that our initial placement may not fully capture its native configuration. Consistent with this ambiguity, a recent ~8 Å cryo-EM reconstruction of the autoinhibited kinesin-1 heterotetramer revealed an asymmetric arrangement of the two KLC molecules spanning the stalk and contacting the motor domains [55]. Given that the TPR domain serves as the primary cargo-binding platform, resolving its conformation in the inhibited state will be essential for developing mechanistic models of how cargo binding releases autoinhibition and initiates motility.

Kinesin-1 has served as the primary model for understanding autoinhibition, but recent studies have begun to define how other kinesin families adopt distinct autoinhibited conformations. Recent work revealed that the kinesin-2 motor harbors a conserved C-terminal β-hairpin motif adjacent to the coiled-coil stalk, which together sequesters the motor domains to prevent microtubule engagement [62–64]. Kinesin-associated protein 3 (Kap3) stabilizes this ‘locked’ state but does not activate the motor; instead, it builds a composite surface where cargo adaptors dock. Cargo binding, such as by Adenomatous Polyposis Coli (APC), displaces the β-hairpin and releases inhibition, enabling motility.

Kinesin-3 motors use a different set of strategies. Most kinesin-3 family members exist as compact, self-inhibited monomers that activate upon dimerization, whereas others can adopt an autoinhibited dimeric state [7]. KIF1C forms a stable dimer in which stalk and forkhead-associated (FHA) domain elements contact the motor domain’s microtubule-binding surface to suppress activity. Cargo adaptors, such as Protein Tyrosine Phosphatase Non-Receptor Type 21 (PTPN21) or Protein Hook homolog 3 (HOOK3), bind the stalk, disrupt the inhibitory interface, and activate motility [65]. HOOK3 binding also unlocks the autoinhibited FTS–HOOK3–FHIP complex to co-recruit dynein–dynactin, enabling co-ordinated bidirectional transport [66]. In contrast, motors such as KLP-6 adopt a compact monomeric inhibited state, in which CC1 and CC2 coils and regulatory domains fold back onto the motor domain to restrain the neck region, stabilize ADP, and block microtubule binding, thereby enforcing a hierarchical ‘multi-lock’ autoinhibition [67].

Together, these studies demonstrate that autoinhibition is a shared regulatory strategy across the kinesin superfamily, albeit achieved through diverse ways. Principles first established in kinesin-1 are now clearly extending across other kinesin families, revealing both shared features and family-specific regulatory mechanisms. Moving forward, a key challenge will be to develop a unified framework that explains how different kinesin classes achieve autoinhibition, how cargo and cofactors unlock activity, and how these mechanisms are tuned to match cellular context and transport demands. Achieving this will be critical for a comprehensive understanding of kinesin regulation and for elucidating how dysregulation of motor control contributes to human disease.

## Part II: *trans*-inhibition of kinesin motility via kinesin-binding protein (KIFBP)

While autoinhibition may serve as the ‘hand brake’ of the kinesin motor, there is a trans-regulator that uniquely binds motor domains directly—KIFBP. KIFBP competes with microtubule binding to inhibit kinesin motility, much like a ‘parking boot’ prevents traffic on the road. Interestingly, KIFBP targets several kinesin families, but not kinesin-1—as we will discuss further—indicating this inhibitor is selective. Therefore, the remainder of this review will shift away from kinesin-1 regulation to discuss the relatively uncharacterized role of KIFBP.

To provide scientific context for KIFBP and address this knowledge gap, we will first discuss the path from initial discovery to assigning KIFBP as a critical regulator of kinesins. Finally, we will summarize a model of kinesin inhibition and look forward to future work needed to understand KIFBP’s *bona fide* role in motor regulation.

### Discovery & physiological relevance

KIFBP was initially identified through two-hybrid screening with the motor domain of the transport kinesin-3 KIF1C [68]. This screen identified a 71.8 kDa, 621 amino acid protein predicted to contain an acidic region followed by two TPR domains, while the remaining ~500 AAs had no identifiable homology models. The prey protein was also observed to bind KIF1B isotypes in cultured fibroblasts, earning it the name KIF1 Binding Protein (KBP/KIFBP) [68]. Excitingly, KIFBP matched an existing hit in the protein database identified earlier in the same year: KIAA1279.

KIAA1279 was first identified in a human brain cDNA library screen [69] and mapped at locus 10q22.1 to encode a protein involved in Goldberg-Shprintzen megacolon syndrome (GOSHS) [70]. GOSHS is a rare autosomal recessive neurodevelopmental disorder characterized by infant onset of microcephaly, facial dysmorphism, intellectual disabilities, and megacolon, and it often co-occurs with Hirschsprung disease [71]. As of 2020, less than 40 cases of GOSHS have been recorded [72,73]. Of these cases, 17 unique point mutations, most of which are nonsense mutations, have been identified, suggesting that KIFBP is functionally crucial for development [70,73–81].

Since KIFBP appeared to be involved in axonal transport and neurodevelopmental disease, several fields took interest in investigating the role of KIFBP. In this part of the review, we will highlight key developmental, biochemical, and biophysical studies that first determined roles for KIFBP in cell development, microtubule dynamic (in)stability, and eventually kinesin inhibition.

### KIFBP is essential for neurodevelopment

As suggested by GOSHS patient phenotypes, KIFBP is an essential neurodevelopmental protein involved in axonal outgrowth. Several groups have characterized loss of KIFBP expression in model organisms and established neurons [78,82–84], showing that lowered KIFBP expression drastically reduces axonal outgrowths by disrupting microtubule dynamics. In zebrafish, an intronic homozygous mutant of KIFBP, *kbp^st23^*, was identified by screening for improper myelination in axons. This mutation results in a truncated KIFBP protein. Zebrafish embryos with *kbp^st23^* mutations showed significant defects in axonal outgrowth as early as 30 hours post-fertilization, but no change in total neuron distribution or morphology compared with WT up to 10 days post-fertilization. These results indicate that KIFBP is essential for axon development, specifically, without affecting neuron differentiation or other major developmental landmarks in zebrafish.

Time-lapse fluorescence microscopy experiments in the *kbp^st23^* zebrafish model revealed axonal shrinkage and reduced growth speed, suggesting that KIFBP may be involved in cytoskeletal dynamics. Transmission EM of fixed *kbp^st23^* zebrafish neurons showed that total axons were reduced and microtubules in the axon were disorganized compared with WT. Mouse neurons electroporated with KIFBP siRNAs showed similar architecture and fate [84]. Defects in microtubule organization, dynamics, and axonal outgrowth are also observed in multiple established neuron lines [78,85]. Together, this work suggests that KIFBP is essential for microtubule organization and dynamicity in neuronal axons during development.

Subsequent work in mouse models showed that loss of KIFBP leads to defects in other organ systems. Homozygous knockout mice had extensive defects not only in brain tissue but also in the respiratory system and gastrointestinal tract, leading to death 4 hours after birth [83]. This evidence suggests KIFBP is a regulator of neuronal and non-neuronal kinesins [85], as we will discuss further.

In summary, KIFBP is required for neuron development, particularly for axonal outgrowth and dendritic branching. Depleting functional KIFBP reduces axon outgrowth by disrupting microtubule organization. This role in microtubule stability is not exclusive to neuron development but likely important for many polarized cell processes, as we will discuss.

### KIFBP regulates cell division

KIFBP also regulates kinesin motors involved in mitosis. For instance, kinesin-binding experiments identified KIFBP as a binder for kinesin-8 (KIF18A) and kinesin-12 (KIF15) motors, both of which are mitotic kinesins [85]. Knockdown of KIFBP in dividing HeLa and RPE1 cells led to improper chromosome alignment and spindle-pole separation [86,87]. Notably, overexpression of KIFBP displaced endogenous KIF18A and KIF15 motors from the mitotic spindle but did not affect another critical mitotic kinesin, Eg5 [86,87]. Furthermore, knockdown of both KIF18A and KIF15 mimics KIFBP overexpression, indicating that KIFBP inhibits both of these mitotic motors in this system [87]. Together, these data suggest that KIFBP directly binds and regulates a kinesin-8 and a kinesin-12 in mitosis.

Interestingly, the co-expression of KIF18A, KIF15, and KIFBP increases the localization of both motors to the mitotic spindle [87]. Malaby et al. hypothesized that while KIFBP interacts directly with the KIF18A and KIF15 motor domains, spindle localization occurs through microtubule binding of the kinesin C-terminal tail. Indeed, C-terminally truncated versions of KIF18A and KIF15 do not localize to the spindle but are diffuse [87]. These results showed that KIF18A and KIF15 can simultaneously bind KIFBP and localize to the spindle. These works provide evidence that a cargo-bound inhibited kinesin motor exists in cells, where the ‘cargo’ are spindle microtubules [86,87]. Crucially, this result also suggests that KIFBP plays a unique role in kinesin inhibition, as autoinhibition of cargo-bound motors has not been observed. Through future work with KIFBP, we may begin to understand fine-tuned trafficking regulation.

Although a direct KIFBP-kinesin interaction is the simplest explanation for the observed phenotypes, it is possible that KIFBP influences microtubule dynamics via interactions with microtubule regulatory proteins. A two-hybrid screen against a mouse cDNA library identified the stathmin SCG10 as a potential KIFBP binding partner [88]. SCG10, as a stathmin, destabilizes microtubules by sequestering tubulin. Like KIFBP, SCG10 promotes neuronal outgrowth during development [89,90]. While their functional roles correlate, KIFBP and SCG10 have not been observed to co-localize in cells or reliably pull down together *in vitro* [85,88]. Therefore, further work is needed to establish a potential interaction between KIFBP and SCG10.

A more robust interaction has been observed between KIFBP and citron kinase (CITK), a midbody cytokinesis factor that interacts with the mitotic kinesin-3 (KIF14) and kinesin-6 (KIF23) [91]. Interestingly, KIFBP can also bind KIF14 [85], introducing potential cross-talk and/or larger protein complexes to this system. CITK directly binds and phosphorylates KIFBP i*n vitro* [91]. No functional significance is known about this phosphorylation event, but it may regulate such cross-talk. KIFBP co-localizes with CITK at the midbody during cytokinesis, and the knockdown of KIFBP in HeLa cells causes significant disorganization of the midbody, cytokinesis failure, and binucleated cells. KIFBP knockdown also increased KIF23 and decreased KIF14 localization at the midbody, implicating a role for KIFBP in mitotic localization once again [91]. While the functional significance of these interactions still remains unclear, KIFBP’s interaction network spans multiple cytoskeletal factors.

In summary, KIFBP is required for proper microtubule arrangement and motor co-ordination in several stages of cell division. At a molecular level, the range of interactions KIFBP utilizes to mediate microtubules is scarcely explored. The limited biochemical and biophysical studies of KIFBP have focused on its direct interaction with kinesin, as we will discuss in the next section.

### KIFBP inhibits kinesins by targeting their motor domains

#### KIFBP selectively regulates several kinesin families

**Figure 2:**
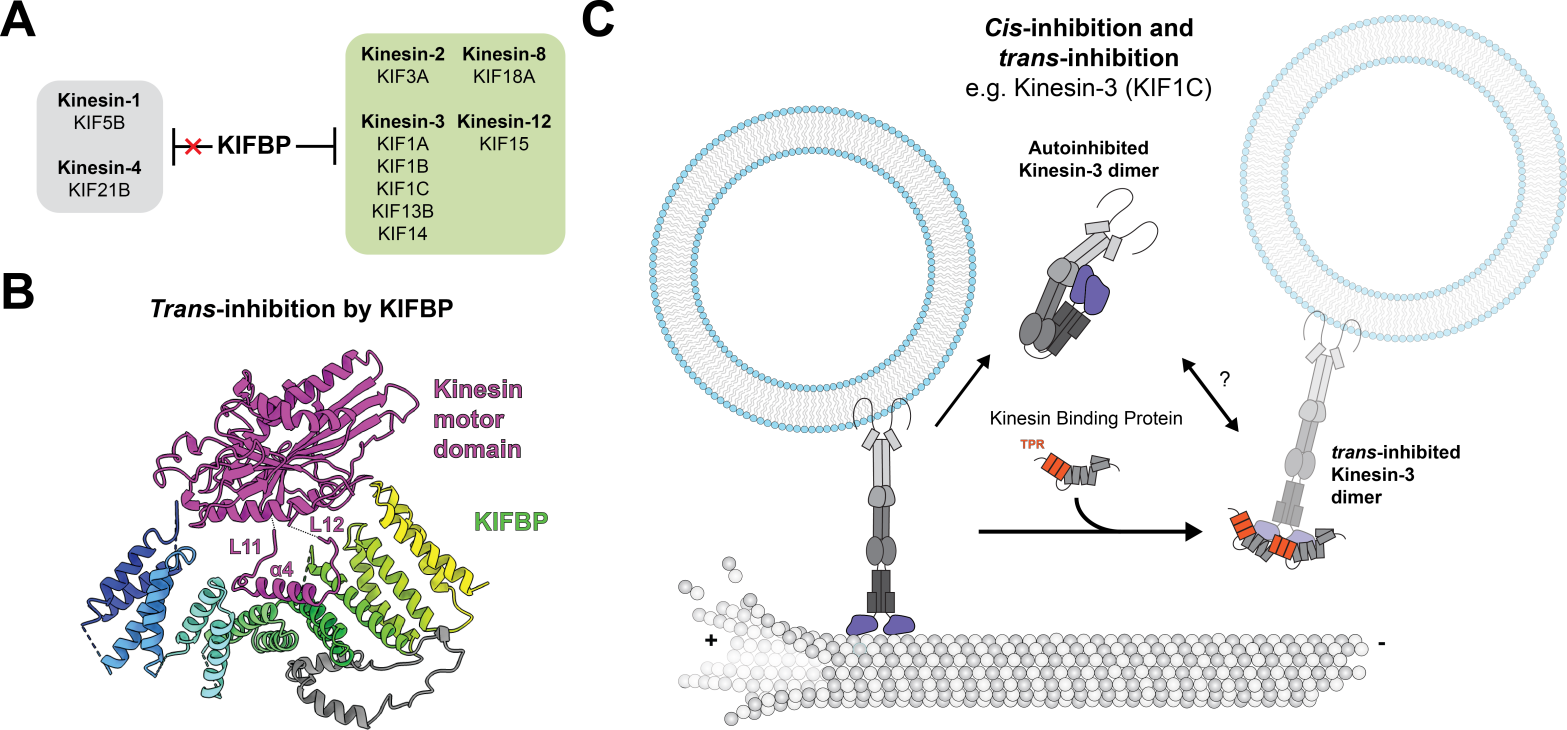
KIFBP is a trans-inhibitor of several kinesins. (**A**) A chart showing which kinesins KIFBP can and cannot inhibit. (**B**) A model of the KIF15 motor domain in complex with KIFBP (PDB: 7RYP). The kinesin α4-helix is remodeled upon binding KIFBP. (**C**) A model of kinesin-3 regulation, which includes both *cis*-inhibition and *trans*-inhibition.

In a landmark biochemical characterization of KIFBP, Kevenaar et al. revealed that KIFBP interacts with many kinesins [85], introducing the idea that KIFBP can interact broadly with diverse kinesin motors. By co-immunoprecipitation (co-IP) mass spectrometry, they showed that KIFBP pulls down kinesins in the kinesin-3 (KIF1A, KIF1B, KIF1C, KIF13B, and KIF14) and kinesin-2 families (KIF3A). Beyond these kinesins, they also found that KIFBP bound to mitotic motors from the kinesin-8 (KIF18A) and kinesin-12 (KIF15) families (Figure 2A). Using a pull-down assay, they verified the interaction of KIFBP with the motors identified in the proximity-based screen and showed that KIFBP could interact with 9 of the 25 motors tested.

Notably, in all of these experiments, Kevenaar et al. did not observe interactions between KIFBP and the kinesin-1 family (KIF5A, KIF5B, KIF5C). This result is significant and unexpected, considering (1) the widespread nature of kinesin-1 across all cell types in the human body and (2) the high degree of conservation for kinesin motor domains. As discussed below, how KIFBP is selective for specific motors remains an area of active investigation.

To understand the effects of KIFBP on kinesin motor activity, Kevenaar et al. used *in vitro* motility assays, microtubule co-pelleting, and rapalog-inducible peroxisome trafficking assays to show that KIFBP inhibits kinesin activity. In one experiment, they added KIFBP to a constitutively active KIF1A motor, showing that KIFBP drastically decreased the landing rate on microtubules and total number of processive events without changing its velocity. This suggests that KIFBP acts as an off-switch rather than a regulator of trafficking speed. In COS7 cells, KIFBP was further shown to inhibit rapalog-induced trafficking by the kinesin-3s KIF1A/B/C and KIF13B, the kinesin-2 KIF3A, the kinesin-12 KIF15, and the kinesin-8 KIF18A. In contrast, KIFBP did not inhibit kinesin-1 KIF5B or kinesin-4 KIF21B in this system (Figure 2A).

Given that KIFBP regulates axonal kinesins and axonal microtubule organization [78,82–84], they next characterized the effects of KIFBP-motor inhibition in cultured dissociated rat neurons. Overexpression of KIFBP or short-hairpin (shRNA) knockdown of the kinesin-8 KIF18A significantly increased microtubule catastrophe and growth rate, while net microtubule length decreased. Conversely, shRNA knockdown of kinesin-3 KIF1A only reduced microtubule growth rate, suggesting that KIFBP mediates microtubule dynamics by regulating multiple kinesin families. Overexpression of KIFBP altered the localization of KIF1A’s cargo Rab3-coated vesicles, indicating that KIFBP regulates anterograde transport of Rab3 vesicles by inhibiting KIF1A. KIFBP knockdown did not affect anterograde trafficking of kinesin-1 cargoes, mitochondria, or autophagosomes, consistent with data that show KIFBP does not interact with KIF5 motors. Surprisingly, the total number of retrograde trafficking events of Rab3 vesicles and mitochondria increased, and retrograde trafficking of autophagosomes decreased, suggesting that KIFBP may co-ordinate bidirectional multi-motor cargo transport. Together, these findings established a library of KIFBP targets, aligned with GOSHS patient presentation and model organisms, and initially detailed a mechanism of action for KIFBP.

#### KIFBP competes with tubulin and remodels the kinesin motor domain

To understand the molecular basis for KIFBP-mediated inhibition of kinesin motor activity, two groups utilized cryo-EM to determine structures of KIFBP alone and KIFBP bound to kinesin motor domains [92,93]. Both our group and Carolyn Moores’ group showed that KIFBP contained a core 4–TPR fold with nine total helical pairs arranged in a right-handed É‘-solenoid. While the overall architecture of KIFBP was identifiable, Atherton et al. were limited to 4.6 Å resolution, and the resulting map was relatively anisotropic due to challenging sample conditions. Due to the lack of structural homologs or accurate structure predictions (this work was completed prior to the release of AlphaFold2), model building into this ambiguous map was challenging. However, Atherton et al. utilized various secondary structure and disorder prediction programs such as TPRpred, iTasser, and Raptor X and homology modeling with HHpred to build fragments into the map from identified homologous structures. Despite these efforts, there was still uncertainty with the map hand and placement of the termini.

Fortunately, the cryo-EM structural analysis from our group pushed the resolution of a core part of KIFBP to below 4 Å, enabling unambiguous identification of side chains for confident model building [93]. To increase the accuracy of model building, we isolated a 3D class of particles from our dataset that lacked several terminal helices but represented a stable region of KIFBP. By further refining this class, we obtained a 3.8 Å resolution map of the KIFBP ‘core.’ *De novo* model building into this map allowed for hand-determination and side-chain assignments. With the orientation of the core determined, we could then fit the rest of the KIFBP backbone into our own 4.6 Å full map, which agreed with Atherton’s map and model.

Beyond showing the overall architecture of KIFBP alone, both publications determined 3D reconstructions of KIFBP bound to kinesin motors. Unexpectedly, the KIFBP-motor structures showed a dramatic remodeling of the kinesin motor domain when bound to KIFBP (Figure 2B). Both papers investigated KIF15 bound to KIFBP, seeing that the motor domain of KIF15 appeared mostly similar to the free motor domain with the exception of the microtubule-binding helix (α4); when the kinesin motor domain was fit into the cryo-EM density, the density for the microtubule-binding α4-helix was missing. Instead, the α4 helix was found ~15 Å away, bound in a hydrophobic groove of KIFBP. This rearrangement may be a conserved mechanism, as we observed the same conformational changes in KIF18A bound to KIFBP [93].

Not only does KIFBP sequester α4 from serving its role as the major tubulin-binding interface, but a rearrangement of α4 likely has drastic consequences on the kinesin mechanochemical cycle. To displace the kinesin α4-helix by 15 Å, part of the kinesin ATPase site is also rearranged (kinesin loop 11, switch-II motif). While published work lacked the resolution needed to determine the nucleotide occupancy in the kinesin motors, we expect future work to show nucleotide-dependent effects of KIFBP on kinesin motors.

The cryo-EM structures of KIFBP bound to KIF15 suggest that KIFBP utilizes multiple interaction points to bind the kinesin motor domain. We extended these structural studies by performing XL-MS of KIFBP-KIF15 [93]. The XL-MS highlighted three distinct regions of KIFBP that interact with the kinesin motor domain, ranging from KIFBP’s N-terminus to the C-terminus. Using mutagenesis and *in vitro* pull-downs, we showed that the N-terminal ‘loop 1’ of KIFBP plays a critical role in binding motors, whereas the middle region ‘loop 14’ only contributes moderately to kinesin binding. Unexpectedly, even though the C-terminus of KIFBP cross-linked strongly to the kinesin motor, we could not identify mutations in this region that disrupt KIFBP motor binding. Continued mutagenesis in the future will help to dissect which parts of KIFBP play essential roles in kinesin recognition and remodeling.

During our structural investigation of KIFBP bound to kinesin motor domains, we could not easily identify structural features that would impart motor specificity for KIFBP (i.e. why does KIFBP bind to KIF15 but not KIF5B). To address this, we leveraged all-atom molecular dynamics simulations of kinesin motor domains alone and compared these results to the motor domains bound to KIFBP. The principal component analysis of the molecular dynamics trajectories showed only KIFBP-binding kinesins occupied distinct regions. These populations represent conformational landscapes unique to KIFBP-binding kinesins, likely contributing to binding specificity. Therefore, we believe structural features intrinsic to kinesin motors enable KIFBP recognition and remodeling, although future work is needed to test this hypothesis further.

### Future of KIFBP

Given the limited research on KIFBP to date, many fundamental questions remain. Below, we highlight some of the most relevant gaps in knowledge regarding kinesin regulation by KIFBP and provide suggestions for a way forward.

What dictates KIFBP selectivity? Currently, we know that KIFBP binds transport kinesins kinesin-2 and kinesin-3, and mitotic kinesin-8 and kinesin-12 family motors but does not bind kinesin-1 (Figure 2A). There is not a high correlation between these specific motors, their cargoes, tissue specificity, or subcellular localization. Furthermore, KIFBP binds along a highly conserved α4 interface that lacks TPR-recognition motifs, such as the KLC W/Y-acidic motif. It is likely that KIFBP recognizes highly specific sequences on kinesin motors. Previously, both our lab and the Moores lab suggested that the differences in KIFBP binders and non-binders lay in the accessory binding domains, where minor changes in the length of kinesin loop 8 could sterically hinder binding [92,93]. However, it remains unclear which exact sequences of the kinesin motor domain mediate accessory binding. We still don’t understand what factors are essential for KIFBP selectivity. Further implementing molecular dynamics simulations to predict conformational landscapes of binders and non-binders could narrow in on more regions of interest. Additionally, pursuing high-resolution cryo-EM reconstructions of KIFBP:kinesin motor complexes could improve model building and clarify how accessory contacts are formed.

The structural analysis showed that KIFBP sterically remodels the kinesin motor domain and blocks microtubule binding. We do not understand why KIFBP remodels α4 in order to block microtubule binding. Further, we do not have measures of KIFBP affinity for the different kinesin motors. One hypothesis would be that fully remodeling a kinesin motor domain would lead to a high-affinity complex of KIFBP-kinesin. Future work is needed to place this interaction in the context of the cell, where structural studies with mutagenesis can help generate tools for cell-based assays.

It remains unclear how KIFBP alters microtubule organization and dynamics. Some of the kinesins implicated as KIFBP binders (e.g. KIF18A) regulate microtubule dynamics [94], suggesting that changes in the activity of KIF18A will alter microtubule organization in cells. Yet, it should be noted that KIFBP may interact with other microtubule regulators and other mitotic kinesins, as discussed [88,91]. Whether microtubule stability is mediated exclusively through kinesin regulation or a more direct interaction remains unknown, but it holds tremendous relevance to understanding GOSHS and fundamental cell biology.

Does KIFBP regulate the trafficking of specific cargoes? Published data show a clear link for KIFBP regulating microtubule organization via kinesin-8 and kinesin-12 [86,87] and Rab3 vesicle trafficking via kinesin-3 [85]. More broadly, we can consider likely cargoes that KIFBP may regulate. For instance, KIFBP may regulate Rab11 recycling endosomes [95], APC vesicles [96], fodrin vesicles [97], NR2B vesicles [98,99], N-cadherin/β-catenin [100], GLI [101], and IFT trains [102] via kinesin-2. Additionally, KIFBP regulates cargoes trafficked by kinesin-3, such as mitochondria [68,82,85]. All of this to say, KIFBP could additionally regulate processes as broad as ciliogenesis, inflammasome signaling, mitochondrial trafficking, and biogenesis [103], and more. As of now, it still remains unclear what kinesin-mediated trafficking events KIFBP actually regulates.

## Part III: updated model of kinesin regulation, from head to tail

In this review, we have highlighted both *cis-* and *trans-*mediated inhibition of kinesin activity. Why have multiple modes of inhibition evolved to regulate kinesin activity? Below, we speculate on why such mechanisms exist and highlight the need for future work to understand the implications of these two modes of inhibition.

Generally, we believe autoinhibition and KIFBP offer the cell two broad scenarios to keep kinesins in an off-state: (1) autoinhibition: kinesins are in an off-state and not bound to cargo; (2) KIFBP: kinesins are in an off-state and could be bound to cargo. As seen over the past several decades, autoinhibited kinesin motors are not associated with either microtubules or cargoes, allowing them to move through the cytoplasm in an off-state (Figure 1C). This freely available kinesin state is likely important for the localization of kinesins in cells and the transport of kinesins by the dynein motor protein complex.

**Figure 3:**
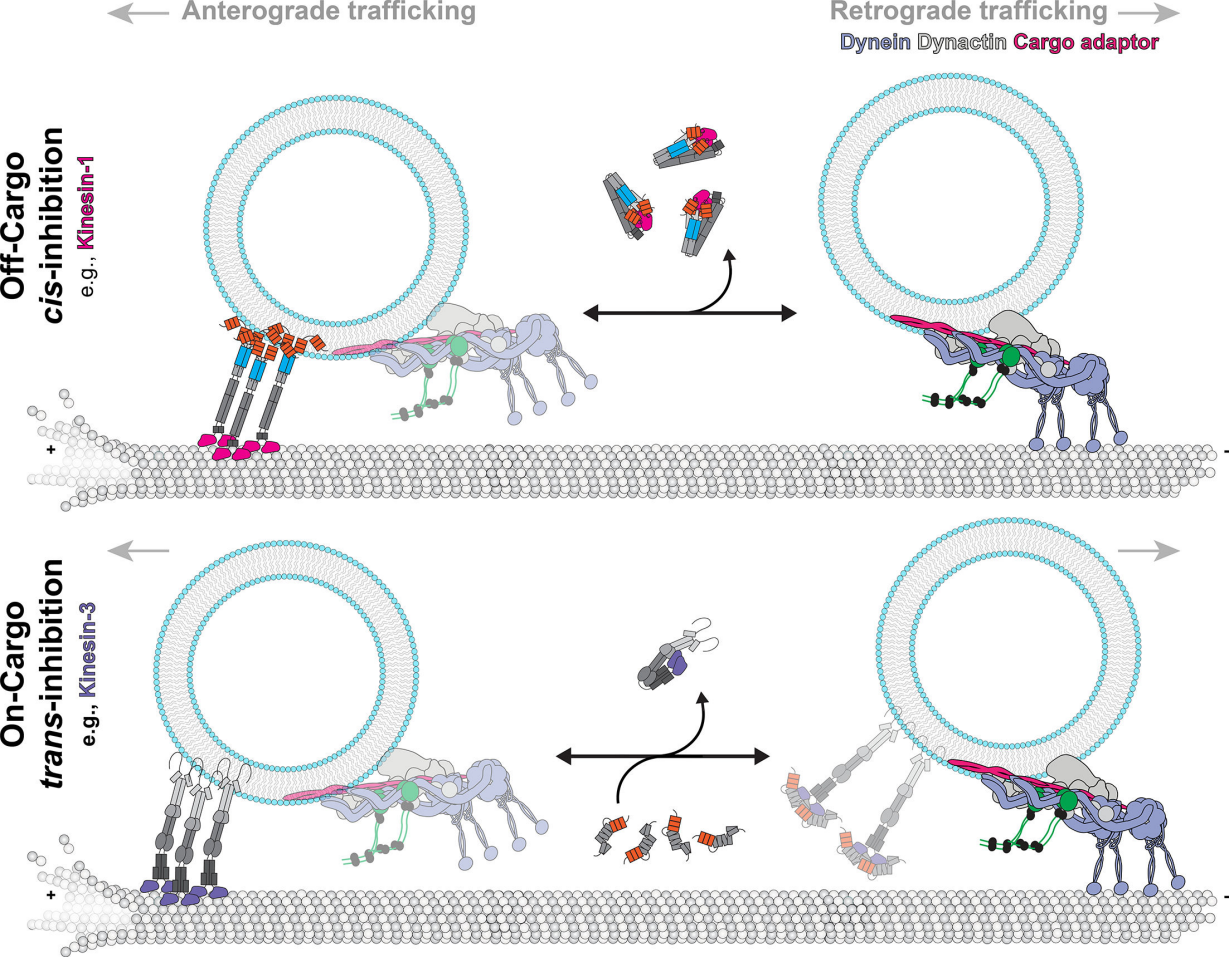
*Cis*-inhibition and *trans*-inhibition of kinesin are distinct regulators of motility. A proposed cartoon model of bidirectional cargo transport. Two forms of regulation are shown: off-cargo *cis*-inhibition and on-cargo *trans*-inhibition. Anterograde transport by kinesin-1 is stopped by *cis*-inhibition, which requires dissociation of the cargo/cargo adapter complex and rearrangement of kinesin light chains. Then, retrograde transport by dynein is promoted (top). We propose that anterograde transport of kinesin-3, for example, is stopped by either *cis*-inhibition or *trans*-inhibition by KIFBP, which creates regulatory layers of unknown functions. In both cases, retrograde transport by dynein is promoted, potentially with kinesin-3 in tow (bottom).

Unlike autoinhibition, KIFBP bound to motor proteins likely offers the cell the ability to keep kinesin motors in an off-state while also enabling the tail of kinesins to bind cargo (Figure 2C). There is little known about when this is used by the cell, but we propose that KIFBP offers a novel ‘cargo-bound off-state’ of kinesin motors, which may be needed during multi-motor transport or to have motors primed for trafficking cargo (Figure 3). This cargo-bound off-state may be advantageous to a recently described system for the KIF1C and dynein co-complex, which is bridged by the adaptor HOOK3 [66]. KIFBP could inhibit KIF1C without disassembling the entire co-complex and deactivating dynein, promoting retrograde transport. Thus, we hypothesize that KIFBP may help to prevent tug-of-war between kinesins and dyneins. During microtubule switching as cargo is transported, it may be advantageous to have a selective inhibitor like KIFBP to turn off populations of kinesins without prolonged stalling or cargo disengagement. Kinesin families are known to co-ordinate with each other to enhance run lengths in multi-motor cargo complexes [104,105]. Therefore, an external regulator, such as KIFBP, could be essential to deliver cargo.

Beyond a role in co-ordinating multi-motor transport, KIFBP may serve as a factor that prevents cargo-bound kinesins from binding to free tubulin. Even though most kinesins have a high affinity for tubulin when incorporated into microtubules, many kinesins bind to free tubulin dimers with moderate affinity [106]. For example, kinesin-3 motor proteins can bind to free tubulin dimers and KIFBP, which suggests that cargo-bound kinesin-3 motors that are not bound to microtubules could affect the soluble tubulin pool (i.e. the ‘tubulin economy’ [107]). Further work is needed to understand if such situations exist in the cell and if KIFBP helps to mitigate any effects of kinesins binding to free tubulin dimers.

Furthermore, it is potentially possible that *cis* and *trans* regulatory interplay exists, where KIFBP binds and regulates auto-inhibited kinesin motors. Currently, there is no evidence that suggests nor denies these layers of inhibition. We expect that future work will explore this relationship and uncover unique functional roles for *cis*-inhibition and *trans-*inhibition.

Finally, we propose that the field should consider KIFBP as a wide-ranging kinesin regulator in a manner analogous to other post-translational modifications (e.g. phosphorylation), offering the ability of a single protein to co-ordinate the activity of dozens of downstream factors. While autoinhibition and cargo/cargo adapter specificity are shared mechanisms across all kinesins, each family has unique interactions required to turn the motor on and off. Most of this variability in regulation occurs at the C-terminus, which we would consider the ‘business end’ of kinesin with regard to cargo transport. However, KIFBP flips the script and takes a more ‘head-on’ approach by binding the motor domain, perhaps bypassing the intricacies of each kinesin family’s regulatory cycle. In this way, KIFBP is a much-needed bridge between different sects of kinesin regulation, allowing the cell to deploy regulation across many kinesin families simultaneously. Future work is needed to map out potential regulatory networks that would employ KIFBP as a tool to globally redirect cargo transport.

## Abbreviations

A: alanine
APC: Adenomatous Polyposis Coli
CITK: citron kinase
EM: electron microscopy
FEZ1: Fasciculation and elongation protein zeta-1
FHA: Forkhead-associated
GOSHS: Goldberg-Shprintzen megacolon syndrome
GRIP1: Glutamate Receptor Interacting Protein 1
HAP1: Huntingtin-associated protein 1
HOOK3: Protein Hook homolog 3
I: isoleucine
JIP1: JNK-interacting protein 1
K: lysine
KAP3: Kinesin-associated protein 3
KHC: kinesin heavy chains
KIFBP: kinesin-binding protein
KLCs: kinesin light chains
MAP7: Microtubule-associated protein 7
MATH: meprin and TRAF homology
MBS: membrane-associated guanylate kinase homolog binding stalk
PTPN21: Protein Tyrosine Phosphatase Non-Receptor Type 21
SEC: size exclusion chromatography
SKIP: SifA and kinesin-interacting protein
TPR: tetratricopeptide repeat
TRAK1: Trafficking kinesin-binding protein 1
WT: wildtype
XL-MS: cross-linking mass spectrometry
cryo-EM: cryo-electron microscopy
